# LNRLMI: Linear neighbour representation for predicting lncRNA‐miRNA interactions

**DOI:** 10.1111/jcmm.14583

**Published:** 2019-09-30

**Authors:** Leon Wong, Yu‐An Huang, Zhu‐Hong You, Zhan‐Heng Chen, Mei‐Yuan Cao

**Affiliations:** ^1^ The Xinjiang Technical Institute of Physics and Chemistry Chinese Academy of Sciences Urumqi China; ^2^ University of Chinese Academy of Sciences Beijing China; ^3^ Department of Computing Hong Kong Polytechnic University Kowloon Hong Kong; ^4^ Guang Dong Polytechnic College Zhaoqing China

**Keywords:** ceRNA network, expression profile, link prediction, lncRNA‐miRNA interaction

## Abstract

LncRNA and miRNA are key molecules in mechanism of competing endogenous RNAs(ceRNA), and their interactions have been discovered with important roles in gene regulation. As supplementary to the identification of lncRNA‐miRNA interactions from CLIP‐seq experiments, in silico prediction can select the most potential candidates for experimental validation. Although developing computational tool for predicting lncRNA‐miRNA interaction is of great importance for deciphering the ceRNA mechanism, little effort has been made towards this direction. In this paper, we propose an approach based on linear neighbour representation to predict lncRNA‐miRNA interactions (LNRLMI). Specifically, we first constructed a bipartite network by combining the known interaction network and similarities based on expression profiles of lncRNAs and miRNAs. Based on such a data integration, linear neighbour representation method was introduced to construct a prediction model. To evaluate the prediction performance of the proposed model, *k‐fold cross validations* were implemented. As a result, LNRLMI yielded the average AUCs of 0.8475 ± 0.0032, 0.8960 ± 0.0015 and 0.9069 ± 0.0014 on *2‐fold*, *5‐fold* and *10‐fold cross validation*, respectively. A series of comparison experiments with other methods were also conducted, and the results showed that our method was feasible and effective to predict lncRNA‐miRNA interactions via a combination of different types of useful side information. It is anticipated that LNRLMI could be a useful tool for predicting non‐coding RNA regulation network that lncRNA and miRNA are involved in.

## 1 INTRODUCTION

Along with the rapid development of molecular biology and bioinformatics, the pattern of coding information in life activity has been found to be far more complex than the genetic central dogma.^1^ ncRNAs (non‐coding RNAs) that are rarely translated into peptides account for roughly up to 98% of total RNAs transcribed by DNA, widely involving in regulating various biological processes such as cancer metastasis, response to stress, development and cell cycle regulation. As two main subclasses of ncRNA, long ncRNAs (lncRNAs) and microRNAs (miRNAs) have attracted great attention for their significant roles in gene expression regulation.^2^, ^3^, ^4^, ^5^, ^6^ miRNAs, as one kind of small ncRNA (20‐25nt), can inhibit translation of mRNA into proteins via mRNA degradation and repressing translation initiation.^7^, ^8^ LncRNAs with lengths of more than 200nt, a loosely classified group of RNA transcripts, can regulate gene expression and nuclear architecture by binding to protein partners via structural motifs as well as interacting with RNA and DNA via base pairing.^9^ Although more and more lncRNAs have been found by computational prediction techniques, improved epigenomic technologies as well as deeper and more sensitive RNA sequencing, only a small number of lncRNAs, like HOTAIR, XIST and TERC, are well studied. It is an urgent need to understand the functional roles and mechanisms of other types of lncRNAs.^9^, ^10^, ^11^

It is reported that lncRNAs are associated with different kinds of biological molecules, forming a complex mechanism by which the expression of proteins is critically regulated.^12^ However, identification of lncRNA‐miRNA interactions based on CLIP‐seq experiments are expensive and time‐consuming for the data collection.^13^ As supplement to biological experimental method, computational methods can combine with other useful information and learn the hidden pattern underlying the known lncRNA‐miRNA interaction network. They are of high efficiency to yield the most potential candidates for experimental validation and therefore attracting increasing attention in the field of non‐coding RNA.

To unify the patterns of different types of non‐coding RNA act in, Salmena et al proposed competing endogenous RNAs (ceRNA) mechanism where different non‐coding RNAs compete for binding to miRNAs that usually repress target gene expression.^1^, ^14^ More and more experimental and theoretical evidence support this hypothesis.^15^, ^16^, ^17^ To annotate the biological functions of lncRNAs, many works have been done to investigate the correlation of expression level between lncRNAs and protein‐coding genes with little consideration on lncRNA‐miRNA interactions.^18^, ^19^, ^20^, ^21^ As the crosstalk between lncRNAs and miRNAs plays a significant role in the biological function, predicting lncRNA‐miRNA interactions by using efficient approaches can contribute to annotating biological functions.^22^

Recent studies show that lncRNAs and miRNAs are involved in the pathological processes of diverse human diseases.^9^, ^23^, ^24^ Therefore, much effort has been made to systematically investigate the impacts of lncRNA‐miRNA interactions. For instance, it is reported that in the vasculature, CERS1, NAT8L and LARP1 as downstream targets can be repressed by the overexpression of miRNAs (miR‐4459, miR4488 and miR‐3960) that bind to lncRNA TGFb2‐OT1 that functions as ceRNA.^25^ Xia et al reported that lncRNA‐FER1L4 in gastric cancer competes for miR‐106a‐5p through the corresponding MREs and then regulates expression of CDKN1A, E2F1, HIPK3, IL‐10, PAK7, PTEN, RB1, RUNX1 and VEGFA.^26^ Du et al investigated into prostate cancer and revealed that, lncRNAs TUG1 and CTB‐89H12.4, acting as miRNA sponges, can suppress tumour and regulate their phosphatase and tensin homolog (PTEN) expression.^27^ Such understanding of regulation network constructed by lncRNAs and miRNAs in pathophysiology can pave the way for new biomarker discovery and therapeutic approaches. However, the number of the existing lncRNA‐miRNA interactions identified by biological experiments is still limited in number.

To accelerate the identification processes of lncRNA‐miRNA interactions, it is an urgent need to propose effective computational methods to find the most potential lncRNA‐miRNA pairs as candidate based on the known interactions.^22^, ^28^, ^29^, ^30^ Most existing computational prediction approaches for miRNA‐target interactions are developed according to some common rules that mainly focus on the following four aspects: conservation, seed match, free energy and site accessibility.^9^ Some prediction tools for miRNA‐target interactions have been proposed. Most of them are based on the observation that the miRNA seed regions of mRNA generally have higher conservation than the non‐seed regions. However, the basic assumption of these methods contradicts the fact that lncRNAs have prominently lower sequence conservation and faster evolution than mRNAs.^31^, ^32^ Some methods are based on the calculation of the free energy of the potential‐binding sites are proposed to predict lncRNA‐RNA interactions.^33^ For instance, LncTar, a prediction tool for lncRNA‐RNA interactions, evaluates the free energy joint structure of each RNA pair.^31^ Although such sequence‐based prediction approaches have been widely applied, they suffer from high false‐positive rates.^28^ Most existing prediction approaches for miRNA‐target interactions are not effective for predicting lncRNA‐miRNA interactions, because such approaches cannot incorporate current understanding of lncRNA‐miRNA interactions.

Previous researches on miRNA‐target threshold effects, small RNA (sRNA) regulation and protein‐protein interaction (PPI) indicate that lncRNAs and miRNAs can interact with each other according to a titration mechanism.^34^, ^35^, ^36^ This finding suggests the importance of expression levels of lncRNA and miRNA on their interaction pattern. In addition, previous study suggests that ceRNA crosstalk is closely related to indirect interactions, the number of MREs, relative abundance of ceRNAs and miRNAs and stoichiometry.^37^, ^38^ More and more studies on co‐expressed gene indicate that associations established by multiple lncRNAs and particular miRNA clusters in a synergistic manner can regulate biological processes.^12^, ^32^

Increasing attention is drawn to predict the interactions between lncRNA and miRNA by considering their general expression patterns. Huang et al, for the first time, use the expression profiles of lncRNA and miRNA to build a computational algorithm called EPLMI predict lncRNA‐miRNA interactions.^39^ This method calculates the final prediction network with the average of two independent prediction networks that are respectively based on expression similarity of lncRNAs and miRNAs. Hu et al proposed an effective prediction model named INLMI that integrated lncRNA/miRNA similarity network was constructed by combining sequence‐based and expression‐based similarity network, and non‐negative matrix factorization (NMF) method was employed on the integrated similarity networks for prediction.^40^

In this work, considering that all lncRNA‐miRNA interactions were positive, we proposed a computational method called LNRLMI to predict potential lncRNA‐miRNA interactions. Specifically, it was based on a constructed lncRNA‐miRNA bipartite network that was composed of similarities of lncRNAs and miRNAs and known lncRNA‐miRNA interaction network. Based on such a constructed network, linear optimization, a semi‐supervise model, was introduced to predict the new links of the known lncRNA‐miRNA interaction network.

To validate the effectiveness of our proposed method, *2‐fold cross validation*, *5‐fold cross validation* and *10‐fold cross validation* were implemented to predict lncRNA‐miRNA interactions on the dataset that was collected from the lncRNASNP database.^41^ LNRLMI was compared with the state‐of‐the‐art computational approaches such as EPLMI and INLMI that were initially developed for predicting lncRNA‐miRNA interactions. Some classical algorithms, such as KATZ measure^42^ and LFM,^43^ were also implemented. Based on the expression profile‐based similarities of lncRNAs and miRNAs, the proposed model yielded the average AUCs of 0.8475 ± 0.0032, 0.8960 ± 0.0015 and 0.9069 ± 0.0014 as well as the best AUCs of 0.8530, 0.9009 and 0.9096 in *2‐fold cross validation*, *5‐fold cross validation* and *10‐fold cross validation*, respectively. The experimental performance of our work illustrates that linear neighbour representation for lncRNA‐miRNA interaction (LNRLMI) is a promising method to predict interactions between lncRNAs and miRNAs.

## 2 MATERIALS AND METHODS

### 2.1 Data processing

To investigate into potential lncRNA‐miRNA interactions, the lncRNASNP database, February 2017 version, is downloaded from http://bioinfo.life.hust.edu.cn/lncRNASNP. The database collected from 108 CLIP‐Seq datasets contains 8091 records of known lncRNA‐miRNA interactions that are confirmed by laboratory studies.^41^ 5348 of valid interactions were obtained for our experiments after data de‐duplication. Specifically, 780 different types of lncRNAs and 275 different types of miRNAs were involved.

Moreover, there are three kinds of similarities of RNAs used as side information in predicting lncRNA‐miRNA interaction. The first type of biological profile is expression profile that is related to human tissues and cell lines. To collect expression profile data, putative functional annotations of lncRNAs and miRNAs were obtained from the NONCODE database (http://www.noncode.org/)^44^ and from the microRNA.org database (http://www.microrna.org/microrna/home.do)^45^, respectively. A total of 450 expression profile data and 264 functional annotations data of the lncRNAs were obtained by converting the names of lncRNA into the NONCODE IDs, in which each expression profile datum of lncRNA has 22 dimensions involved in 8 cell lines and 16 distinct human tissues. And 230 of expression profile data of miRNAs were obtained, which have 172 dimensions, each of which describes the expression level in a specific human tissue or cell line. The second type of similarity is biological function that is related to functional annotations and interactions between target genes. The data are downloaded from miRTarBase (version 6.1, http://miRTarBase.mbc.nctu.edu.tw)^46^, and 272 records of miRNAs in our dataset were collected. Lnc‐GFP method based on a coding‐non‐coding co‐expression network was employed to predict probable biological functions, and 10 most of probable biological function are predicted as the functional annotations for lncRNAs. The third type of information is RNA sequence that is collected from the miRBase database (http://www.mirbase.org/)^47^ and LNCipedia database (https://lncipedia.org/)^10^.

These three types of biological information are widely used in bioinformatics researches. We considered these three types of side information are closely related, and therefore they collectively describe the relation of different types of lncRNA/miRNA with regards to their roles in the regulation network.

### 2.2 Construction of lncRNA/miRNA similarity

In the proposed model of LNRLMI, similarity matrixes are needed to be computed by using different types of raw features of lncRNA and miRNA to search the neighbours that are correlated with regards to lncRNA‐miRNA interactions. In this work, three kinds of similarities were explored to predict lncRNA‐miRNA interactions. The similarity matrix of expression profile of RNAs is computed by using Pearson correlation coefficient (PCC),^48^ which is widely used to depict correlation coefficient of two samples with same type of attributes as follow:

(1) $$\text{PCCS(}X,Y)=\frac{\sum_{i=1}^{N}\left(X_{i}-\overset{¯}{X}\right)\left(Y_{i}-\overset{¯}{Y}\right)}{\sqrt{\sum_{i=1}^{N}{\left(X_{i}-\overset{¯}{X}\right)}^{2}\sum_{i=1}^{N}{\left(Y_{i}-\overset{¯}{Y}\right)}^{2}}}$$

where *X* and *Y* denote the samples with distinct attribute vector that contains *N* attribute value. *X_i_* and *Y_i_* denote the *i*‐th attribute value. PCC score lies between −1 and 1 where maximum and minimum value denotes the strong positive correlation and negative correlation.

For the second type of similarity, given two functional annotations of lncRNA/miRNA(say *R_a_* and *R_b_*), the definition of functional similarity measure is as follow:

(2) $$\text{FNS(}r_{a},r_{b})=\frac{\text{card}(R_{a}\cap R_{b})}{\sqrt{\text{card(}R_{a})}\cdot \sqrt{\text{card(}R_{b})}}$$

To calculate the third type of similarity, the Needleman‐Wunsch pairwise sequence alignment is employed on the sequence data of RNAs via the package of *pairwise2* in *Biopython*. In detail, gap‐open extending penalty, gap‐open penalty and identification score were set as <0.1, −0.5 and 2, respectively.

To incorporate our proposed prediction model, similarity matrixes should be normalized from 0 to 1 if elements of matrix do not range from 0 to 1. In detail, sequence‐based similarities need to be normalized. Elements of each column are divided by the maximum in each column. The final matrix is obtained by filling the upper triangular matrix with the transpose of the lower triangular.

### 2.3 Linear neighbour representation method for predicting lncRNA‐miRNA interactions

**Figure 1:**
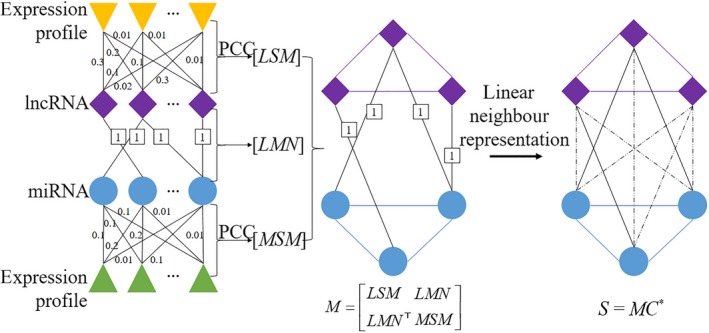
The flowchart of prediction process of LNRLMI

In this section, we propose a linear neighbour representation method for predicting lncRNA‐miRNA interactions (see Figure 1).^49^

Based on an assumption that lncRNAs with similar functions tend to interact with functionally similar miRNAs and vice versa, similarities of RNAs can be helpful information to reflect the correlation between RNAs. As it is reported that the interactions between lncRNA and miRNA could be affected by their expression pattern, it could be expected that their underlying biological function is closely related to their expression pattern. In the framework of our proposed algorithm, a bipartite network is constructed with the similarities and known interaction network. Note that the bipartite network is a real symmetric matrix. All elements of each row can contribute to linear neighbour representation in training. In order to validate the effectiveness of using side information, our proposed method was also employed on a single‐layer network without using side information. Meanwhile, the results can justify the assumption that lncRNAs with similar functions tend to interact with functionally similar miRNAs.

In the framework of our proposed method, first, similarity matrixes of lncRNA and miRNA are constructed, that is, lncRNA similarity matrix $LSM\in \;R^{ln\times ln}$ and miRNA similarity matrix $MSM\in \;R^{mn\times mn}$. The known lncRNA‐miRNA interaction network $LMN\in R^{ln\times mn}$ is then constructed based on the known interaction pairs of lncRNA and miRNA. A target lncRNA‐miRNA bipartite network $M\in R^{N\times N}$ is constructed by combining *LSM*, *MSM* and *LMN* and defined as follow:

(3) $$M=\left[\begin{array}{c} \mathrm{LSM}\\ LMN^{T} \end{array}\right.\left.\begin{array}{c} \mathrm{LMN}\\ MSM \end{array}\right]$$

where *M* can also be treated as a weighted graph *G*(*V, E, W*) that *V*, *E*, *W* denote the vertices, edges and weights, respectively. Note that *E* and *W* are respectively related to *LMN* and similarity matrixes of *LSM* and *MSM*. The corresponding link *m_ij_* is defined as a weighted link form node *i* to node *j*. Here, a score matrix *S* is defined as follows:

(4) S=MC

where *C* is a weight matrix. Specifically, denote an element *s_ij_* in *S*, and each element can be unfolded by a linear summation of contributions from node *i*'s neighbours, as follows:

(5) $$s_{\mathrm{ij}}=\sum_{k}m_{\mathrm{ik}}c_{\mathrm{kj}}$$

where *c_kj_* is the contribution from node *k* to node *j*. In the score matrix S, the observed links are utilized to estimate the rationality of *S*, and the non‐observed ones are undetermined and need to be predicted. According to self‐consistence, the value of *m_ij_* has obviously positive correlation with the score of *s_ij_*, and *S* is closely related to *M* so the magnitude of *C* should be small. Thus, to obtain the matrix *S*, *C* can be simply obtained by solving optimization problem as follows:

(6) $$\min_{C}\alpha \left|\right|M-MC\left|\right|+\left|\right|C\left|\right|$$

where parameter *α* is set to balance the two factors and ||·|| is defined as a certain matrix norm. To solve Eq. (6), the Frobenius norm is used and set with power 2. That is to optimize the minimum of the following formula:

(7) $$Q=\alpha \left|\right|M-MC{\left|\right|}_{F}^{2}+\left|\right|C{\left|\right|}_{F}^{2}$$

where the function $\left|\right|\cdot \;{\left|\right|}_{F}^{2}$ can be solve as $||A{\left|\right|}_{F}^{2}=\text{Tr(}A^{T}A)$. Eq. (7) can be unfolded as follows:

(8) $$\begin{array}{c} Q=\alpha \text{Tr}\left[{\left(M-MC\right)}^{T}\left(M-MC\right)\right]\;\text{+ Tr}\left(C^{T}C\right)\\ =\alpha \text{Tr}\left(M^{T}M-M^{T}MC-C^{T}M^{T}M+C^{T}M^{T}MC\right)+\text{Tr}\left(C^{T}C\right) \end{array}$$

then take partial derivative of *Q* with respect to *C* as follows:

(9) $$\frac{\partial Q}{\partial C}=\alpha \left(-2M^{T}M+2M^{T}MC\right)+2C$$

Taking Eq.(9) as 0, the optimal solution of *C* can be obtain as follows:

(10) $$C^{*}=\alpha {\left(\alpha M^{T}M+E\right)}^{-1}M^{T}M$$

where *E* is the identity matrix. The final score matrix *S* for link prediction can be solved as follows:

(11) $$S=MC^{*}$$

Finally, the target prediction network is computed as $LMN^{\prime }$ in *S*.

## 3 RESULTS

### 3.1 Performance evaluation using k‐fold cross validation

To evaluate the performance of LNRLMI, *k‐fold cross validations* were employed and corresponding receiver‐operating characteristics (ROCs) curves were drawn. In addition, area under curve (AUC) values lying between 0.5 and 1 are calculated to measure whether models perform well or not. AUC of 0.5 denotes a simply random prediction and AUC of 1 denotes an ideal prediction. We implemented 2‐fold, 5‐fold and 10 fold cross validation to investigate how the quantity of training sample influence the performance and evaluate the performance better.

According to *5‐fold cross validation*, the known lncRNA‐miRNA samples were randomly divided into five parts, in which four of them take turn to be used to train the model and the rest one is for testing. To avoid the bias brought by random sample division, random sampling was carried out for 20 times. Consequently, our proposed method yielded good performance with average AUC of 0.8960 ± 0.0.0015 and the highest AUC of 0.9009.

**Figure 2:**
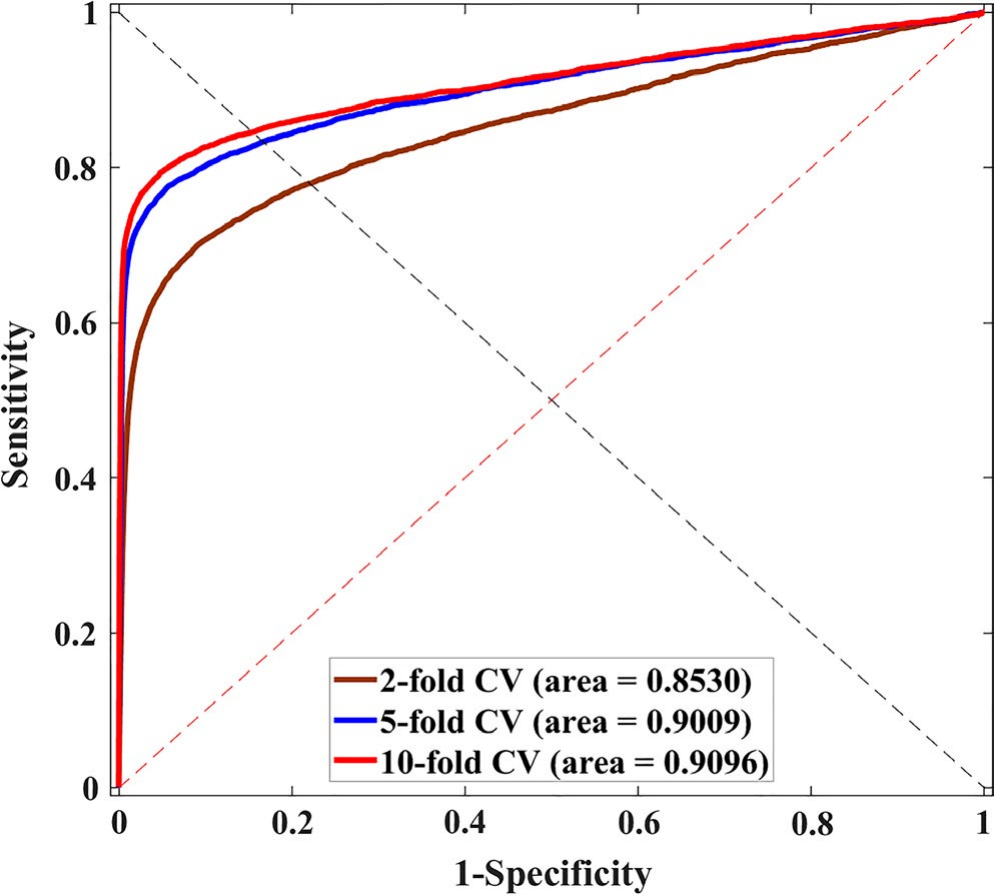
Performance results of LNRLMI by using *2‐fold*, *5‐fold*, *10‐fold cross validation*

We also performed *2‐fold* and *10‐fold cross validation* for further performance evaluation. As a result, the model proposed average AUCs of 0.8475 ± 0.0032 and 0.9069 ± 0.0014 with the highest AUCs as 0.8530 and 0.9096 when performing *2‐fold cross validation* and *10‐fold cross validation*, respectively (see Figure 2). The result demonstrates that our proposed method is effective to predict lncRNA‐miRNA interactions on a large scale.

### 3.2 Evaluation on the effectiveness of using side information

**Figure 3:**
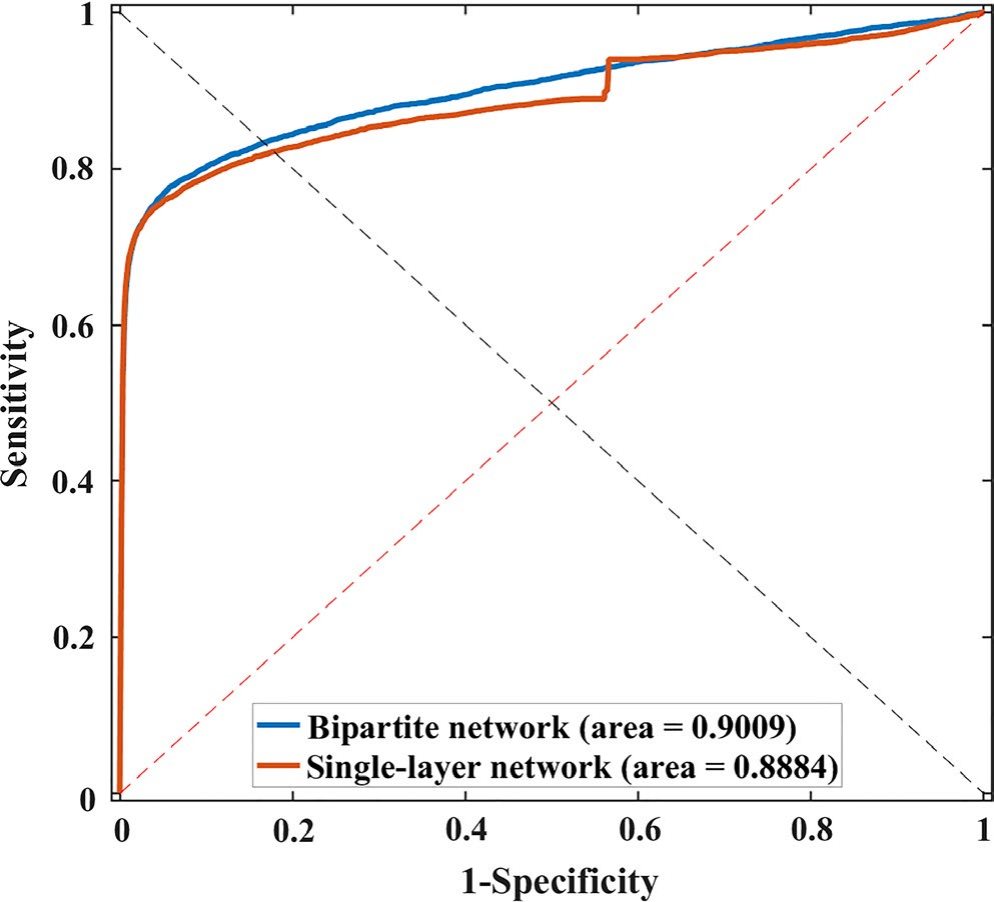
Performance results of LNRLMI by using bipartite network and single‐layer network

Based on the assumption that lncRNAs with similar profile tend to interact with same miRNAs, we here implemented *5‐fold cross validation* on a bipartite network combining with expression‐based similarities and known interaction network as well as a single‐layer network without any side information for 20 times, respectively. As a result, the highest AUC of 0.8884 and average AUC of 0.8838 ± 0.0017 were achieved without using side information while the highest AUC of 0.9009 was yielded by using expression profile‐based similarity. The ROCs of best performance were also plotted in Figure 3.

From the results, the assumption was justified and the biological similarity as side information in prediction model could improve the performance.

### 3.3 Comparison with different kinds of side information

In this sub‐section, other kinds of bio‐information were also investigated, such as nucleotide sequence information derived from high‐throughput sequencing and biological functional information. Two types of similarity were constructed by using sequence data and biological functional data, respectively.

For the purpose of comparison with the performance achieved by using expression similarity of RNAs, we similarly employed *5‐fold cross validation* on using biological functional similarity and sequence similarity, respectively. As a result, the average AUCs of 0.8940 ± 0.0019 and 0.8970 ± 0.0017 were yielded by using function‐based similarity and sequence‐based similarity, respectively. The best performance was achieved at AUC of 0.8980 (function‐based similarity), 0.9007 (sequence‐based similarity) and 0.9009 (expression profile‐based similarity). By using expression profile‐based similarity, it reached the lowest standard deviation that demonstrate the better stability. All the results were shown in Table 1. From all results, the performance among three types of similarities were close, which releases that the model might make full use of the side information.

**Table 1:** Performance comparison among different kinds of similarity with regards to AUC values

ncRNA similarity	*2‐fold* CV result	*5‐fold* CV result	*10‐fold* CV result
Expression profile‐based	0.8475 ± 0.0032	0.8960 ± 0.0015	0.9069 ± 0.0014
Sequence‐based	0.8522 ± 0.0034	**0.8970 ± 0.0017**	**0.9070 ± 0.0017**
Function‐based	**0.8523 ± 0.0032**	0.8940 ± 0.0019	0.9039 ± 0.0016

The highest AUCs of *k‐fold* CV by using different kind of similarity is in bold

The results yielded by respectively using three kinds of similarity showed that the side information was helpful to yield a better result.

### 3.4 Comparison with different prediction methods

To evaluate the performance of our proposed method, we compared it with current state‐of‐the‐art computational methods based on the same similarities of lncRNA and miRNA (see Table 2). KATZ measure, a graph‐based computational method, is proposed to solve link prediction problem by computing similarities between nodes and is widely used in social network and biological network. As such prediction task can be tackled by using matrix completion method, LFM was implemented. We also compared our proposed method with the‐state‐of‐the‐art methods such as EPLMI and INLMI that were previously developed for predicting lncRNA‐miRNA interactions. As the first effective technique to predict potential links in the bipartite graph, EPLMI combined two outputs that were based on lncRNA and miRNA by using the two‐way diffusion method. INLMI integrated expression profile‐based similarity and sequence‐based similarity and employed NFM method and two‐way diffusion method to obtain the prediction results.

**Table 2:** Performance comparison among different methods

Method	KATZ^42^	LFM^43^	EPLMI^39^	INLMI^40^	LNRLMI
AUC	0.7439 ± 0.0017	0.8253 ± 0.0024	0.8447 ± 0.0017	0.8517	**0.8960 ± 0.0015**

Compared with different prediction methods, our proposed method achieved the highest AUC that is shown in bold

As a result, LNRLMI model yielded the highest AUC among other five methods when using *5‐fold cross validation*. INLMI, EPLMI, KATZ and LFM yielded AUCs of 0.8517, 0.8402, 0.7435 and 0.8257, respectively. With comparison to other algorithms, LNRLMI is considered as a reliable and promising tool to predict lncRNA‐miRNA interactions on a large scale.

### 3.5 Sensitivity to hyper‐parameter

**Figure 4:**
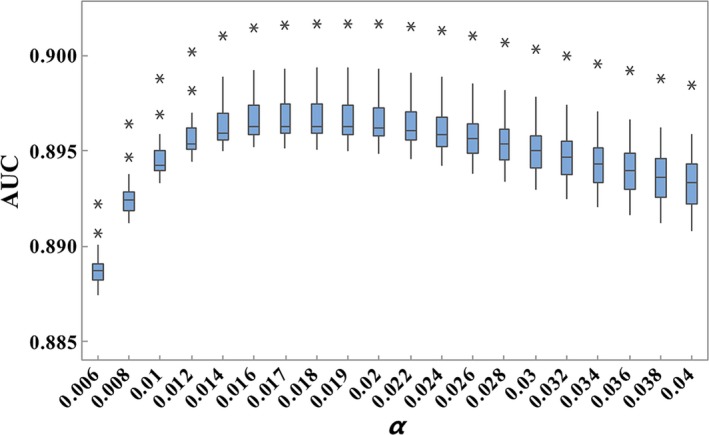
Performance results of different parameter *α*

Our proposed method has one hyper‐parameter *α*, where *α* can balance two factors in solving optimization problem. We studied the sensitivity of *α* by ranging it from 0.006 to 0.04 at an interval of 0.002. We tested the performance by implementing each experiment at different parameter *α* for 20 times. As a result, the highest AUC was yielded, when *α* was 0.018. To search the best parameter, *α* of 0.017 and 0.019 were also tested. From Figure 4, the best performance was achieved with *α* of 0.018. The box plot in Figure 4 shows that the distribution of AUC is on a bell‐shape curve, which demonstrates that the proposed model could be easily optimized. In addition, the prediction performance tends to be stable with AUCs of around 0.895 when *α* increases up to 0.014. Therefore, we consider the proposed model is robust to the setting of hyper‐parameter, which is important for its application on various and large datasets.

## 4 DISCUSSION

In this work, we aimed to develop a robust method to investigate into the potential lncRNA‐miRNA interaction from the current limited data. First, we constructed a bipartite network by combining known lncRNA‐miRNA interaction network as well as expression profiles‐based similarities of lncRNA and miRNA via PPC method. Then, LNRLMI prediction model was built to predict potential interactions between lncRNAs and miRNAs, based on the linear neighbour representation of complex network. In order to validate the effectiveness and reliability, we implemented *k‐fold cross validation* and compared it with recent state‐of‐the‐art methods. As a result, our proposed method yielded the highest AUC score of 0.8999 in *5‐fold cross validation* among five methods. In addition, we also implemented *2‐fold cross validation* and *10‐fold cross validation* that demonstrated that our proposed method could yield a better result if a larger number of training samples are introduced into the model.

We anticipate that LNRLMI can offer great insights into the mechanism of ceRNA regulation networks that lncRNA and miRNA are involved in. Different from traditional prediction tools that focus on binding sites, LNRLMI are only based on the network structure of lncRNA‐miRNA interactions with node attributes. As we correspondingly defined the model as a semi‐supervised one, it is to fill the matrix with prediction scores for all lncRNA‐miRNA pair candidate and doesn't need any negative sample. In the process of LNRLMI, the interaction possibility between lncRNAs and miRNAs can be yielded by using the expression similarity of lncRNA and miRNA in one‐shot, which is more effective than the former method.

In the experimental comparisons, there are some points that should be noted: (a) using a bipartite network can perform better than using a single known interaction network, which demonstrates the side information is meaningful in the prediction model; (b) in cross validation experiments, more data on lncRNA‐miRNA interactions for training can yield a better performance, demonstrating that the more precise result can be obtained by offering more complete network; (c) By using different types of similarity, the AUCs were close, which demonstrated that the constructed model made full use of the side information and reached the best performance. (d) Compared with the exiting methods that EMPLMI and INLMI employed two‐way diffusion without considering weighting, LNRLMI is more effective and reliable to yield prediction results of lncRNA‐miRNA interactions by using the bipartite network that combines the expression similarities of lncRNA and miRNA as well as the known lncRNA‐miRNA interaction network.

Even though LNRLMI is effective and reliable as demonstrated by the experimental results, some of its limitations should be noted. Imbalanced data amounts of sample number for different lncRNA/miRNA might result in prediction‐bias. Moreover, if lncRNA/miRNA are well studied further, better prediction results can be yielded owing to a more complete lncRNA‐miRNA interaction network.

## CONFLICT OF INTEREST

The authors declare that they have no conflict of interest.

## AUTHOR CONTRIBUTION

LW conceived the project, developed the prediction method, designed the experiments, analysed the result and wrote the manuscript. YAH implemented the experiments, analysed the result and revised the manuscript. ZHY and ZHC analysed the result and revised the manuscript. MYC analysed the result. All authors read and approved the manuscript.

## Data Availability

The data that support the findings of this study are available in [lncRNASNP, NONCODE, The microRNA.org resource, miRTarBase, miRBase, LNCipedia] at [10.1093/nar/gku1000; 10.1093/nar/gkr1175; 10.1093/nar/gkm995; 10.1093/nar/gkv1258; 10.1093/nar/gkt1181; 10.1093/nar/gks915], reference number.^10^, ^41^, ^44^, ^45^, ^46^, ^47^ These data were derived from the following resources available in the public domain:http://bioinfo.life.hust.edu.cn/lncRNASNP; http://www.noncode.org; http://www.microrna.org/microrna/home.do; http://miRTarBase.mbc.nctu.edu.tw; http://www.mirbase.org/index.shtml; https://lncipedia.org/.
